# Reducing Complications in Pancreaticoduodenectomy

**DOI:** 10.3390/cancers18040630

**Published:** 2026-02-14

**Authors:** Josh B. Karpes, Ken Liu, Michael D. Crawford, Carlo Pulitano, Charbel Sandroussi, Jerome M. Laurence

**Affiliations:** 1 Department of Upper Gastrointestinal and Hepatobiliary Surgery, Royal Prince Alfred Hospital, Camperdown, Sydney 2050, Australia; 2 Australian National LT Unit, Royal Prince Alfred Hospital, Sydney 2050, Australia; 3 Sydney Medical School, University of Sydney, Sydney 2006, Australia; 4 RPA Institute of Academic Surgery, University of Sydney, Sydney 2006, Australia

**Keywords:** pancreaticoduodenectomy, postoperative pancreatic fistula, centralisation, failure to rescue

## Abstract

Pancreatic surgery is a technically demanding field associated with frequent morbidity, with pancreatic fistula representing the dominant driver of major complications in pancreaticoduodenectomy (PD). Although refinements in operative technique, perioperative management, and institutional systems have contributed to incremental improvements, the overall incidence of clinically relevant complications has remained largely unchanged over recent decades. This narrative review provides a comprehensive overview of current strategies aimed at reducing morbidity and mortality after pancreaticoduodenectomy, focusing on modifiable technical, pharmacological, nutritional, and systems-based interventions, whilst acknowledging the underlying biological determinants that remain difficult to alter. This review synthesises contemporary evidence on fistula risk modelling, anastomotic reconstruction, and adjunctive operative techniques. The role of pharmacological interventions is examined alongside an evaluation of perioperative nutritional optimisation and enhanced recovery frameworks. Systems-based strategies such as centralisation, failure-to-rescue performance, protocolised pathways, and algorithm-driven postoperative surveillance are highlighted as emerging areas with substantial potential to impact survival independently of complication rates. Finally, this review explores future directions, including radiomics-based risk stratification, intraoperative imaging, and tailored postoperative care. Together, these domains provide a platform for reducing complication severity, standardising postoperative care, and ultimately improving patient outcomes. By integrating these perspectives, this review aims to present a comprehensive and in-depth narrative of how to reduce complications in pancreas surgery. Overall, this narrative review proposes that meaningful improvements in outcomes after PD likely do not arise from the elimination of complications altogether, but rather from improved prediction, prevention where possible, and critically, more effective systems of care that reduce the severity and consequences of complications when they occur.

## Simple Summary

Pancreatic surgery is one of the most complex areas of abdominal surgery, with morbidity and mortality remaining a major challenge. Despite progress in surgical techniques and perioperative care, outcomes still vary widely, and there is no consensus on how to reliably prevent major complications. This study evaluates contemporary evidence on how complications develop, how they can be detected early, and the strategies that may reduce their frequency and impact. The evaluation includes technical factors during surgery, as well as non-technical factors outside of the operating theatre that may improve safety and outcomes. The goal of this review is to guide practice and future research to improve the safety of pancreatic resection in any environment.

## 1. Introduction

Pancreaticoduodenectomy remains among the most complex operations in abdominal surgery, performed predominantly for malignant and pre-malignant conditions of the pancreas and the periampullary region. Despite substantial advances in surgical technique, imaging, perioperative care, and multidisciplinary oncological management, morbidity after pancreatic surgery remains high. Postoperative pancreatic fistula (POPF) continues to be the most important driver of adverse outcomes, contributing to infection, haemorrhage, delayed gastric emptying, prolonged admission, and mortality.

The published literature reflects a heterogenous array of approaches to reduce risk of complications, ranging from technical modifications of pancreatico-enteric anastomosis to the selective use of somatostatin analogues, enhanced nutritional support, and structured institutional pathways. However, significant variation persists between centres, and no single strategy reliably prevents POPF across all patient populations. Concurrently, broader health system factors such as volume, rescue capability, and adherence to protocols in postoperative care have emerged as key determinants of survival, suggesting that outcomes depend not only on the prevention of complications but also on the systems in place to recognise and manage them.

Given the complexity of PD and the underlying multifactorial nature of its complications, an integrated review of technical, pharmacological, nutritional, and systems-based strategies is essential. This narrative review aims to evaluate the modifiable factors influencing postoperative outcomes, highlight areas of consensus and controversy, and propose future directions for research and clinical practice. Through this synthesis, we aim to clarify the current state of the field and support ongoing efforts to improve the safety and effectiveness of pancreatic surgery in general, and more specifically, pancreaticoduodenectomy.

## 2. Methodology

**Figure 1:**
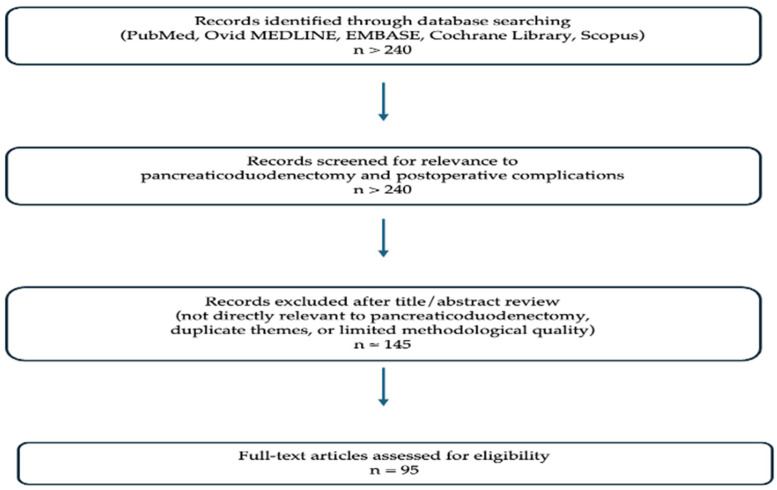
Flow diagram of study selection.

A structured literature search was undertaken with the intent of conducting a narrative review across major electronic databases, including PubMed, Ovid Medline, EMBASE, the Cochrane Library, and Scopus, with the earliest year of publication set at 1990. Only studies published in English were included. Search terms encompassed “pancreaticoduodenectomy,” “postoperative pancreatic fistula,” “risk scores,” “anastomosis,” “pharmacological prophylaxis,” “perioperative nutrition,” “centralisation,” “failure to rescue,” and “algorithm-based care.” Randomised controlled trials, cohort studies and large registry analyses, systematic reviews and meta-analyses, international consensus statements, and landmark observational studies were considered. Given the heterogeneity of study designs and outcomes, a narrative review was planned from the outset. Following the initial search, more than 240 studies were identified as relevant to the scope of this review, with 95 selected for detailed evaluation and inclusion (See Figure 1 for flow diagram of study selection). Reference lists of key articles were also screened to identify additional relevant studies. This narrative review draws primarily on higher-level evidence, including randomised trials, systematic reviews and meta-analyses, and consensus guidelines, supplemented by carefully selected observational studies where higher-level data were limited. Studies were purposively selected based on level of evidence, methodological quality, recency, and direct relevance to strategies aimed at reducing complications after pancreaticoduodenectomy, with emphasis on modifiable and non-modifiable factors, technical approaches, perioperative interventions, and systems-based strategies.

## 3. Postoperative Pancreatic Fistula—A Key Driver of Morbidity

Postoperative pancreatic fistula represents the most consequential complication following pancreaticoduodenectomy and is a major determinant of prolonged recovery, failure to rescue, and overall postoperative morbidity and mortality. The original definition of POPF was agreed upon in 2005 by The International Study Group on Pancreatic Surgery (ISGPS), describing an “abnormal communication between the pancreatic ductal epithelium and another epithelial surface containing pancreas-derived, enzyme-rich fluid” [1]. To clinically define this, amylase from an operatively placed drain that was greater than three times the upper limit of the serum value was classified as a pancreatic fistula. This was further graded into A, B, or C depending on severity. This classification was then revised in 2016 with the goal of improving the universal consistency in evaluating the clinical outcomes of pancreatic fistulas, with Grade A considered a “biochemical leak,” whereas B and C changed to “clinically relevant postoperative fistula.” The clinically relevant leaks either required some form of change in management, rendered a patient in need of another operation, or caused organ dysfunction [2].

Pancreas cancer, one of the primary indications for pancreaticoduodenectomy, carries a global incidence of approximately 8.14 cases per 100,000 person-years, reflecting a devastating problem worldwide and demonstrating the importance of reducing complications in the only known curative method [3]. However, despite advances in technology, surgical technique, and resources, the incidence of POPF has not changed over the last few decades, presumably due to an incomplete understanding of the underlying pathological processes [4]. At the time of the revised ISGPS guidelines in 2016, the incidence of POPF was quoted as ranging between three and forty-five percent of pancreatic operations at high-volume centres [2], with more recent data reporting almost identical numbers, with one study suggesting that up to forty-one percent of all pancreatic resections will result in a fistula to some extent [5], and another reporting a range of five to fifty-two percent across different institutions [6]. When accounting for clinically relevant fistulas only, one systematic review and meta-analysis found that the incidence remained as high as 17% [7], indicating a substantial burden in the postoperative course of pancreatic resections. Furthermore, there are suggestions from some studies that the risk of death doubles in those patients who develop a POPF [8].

The pathogenesis of POPF following pancreaticoduodenectomy is believed to result from growth failure in the pancreatic anastomosis, consequently leading to the leakage of pancreatic enzymes into the peritoneal cavity [9,10]. The biological basis of this is hypothesised to arise from intrinsic gland characteristics of the pancreas [7]. A soft, non-fibrotic pancreas with high acinar cell density produces abundant exocrine secretions and results in a potentiated inflammatory cascade, which in turn has been associated with higher rates of postoperative pancreatic fistula, as well as higher grades of severity [11,12,13]. Another intrinsic gland characteristic associated with POPF is the duct diameter; a duct of less than three millimetres has been associated with higher rates of fistula due to the technical challenge of creating a secure anastomosis, as well as the resulting inflammation-related duct constriction and subsequent intraductal hypertension [14,15,16]. Furthermore, remnant ischaemia resulting from compromised microvascular perfusion at the anastomotic site may further impair healing [4], highlighting that POPF represents a multifactorial process involving biochemical output, gland texture, ductal and parenchymal quality, and vascular supply. Table 1 outlines the non-modifiable and modifiable factors associated with prediction of POPF.

Whilst POPF remains the dominant driver of morbidity after PD, other clinically relevant complications also warrant consideration. Delayed gastric emptying is common and multifactorial in pathogenesis, influenced by reconstruction type, postoperative inflammation, and pyloric dysfunction, and can prolong hospitalisation [17]. Post-pancreatectomy haemorrhage, despite being less frequent, is associated with significant mortality and often reflects sentinel bleeding from anastomotic breakdown, pseudoaneurysm formation, or infection [18]. Therefore, prompt recognition and access to interventional radiology are critical. Infectious complications independent of fistula, including surgical-site infections, intra-abdominal collections, and cholangitis, are also important determinants of recovery and are influenced by antimicrobial strategy, drain management, and perioperative nutrition. Together, these complications highlight that improving outcomes after PD requires a holistic approach that extends beyond fistula prevention alone.

## 4. Technical Strategies

As POPF remains the principal driver of morbidity after pancreatic resections, technical strategies around anastomosis must play an important role in attempts to reduce its incidence and consequences. However, the biological predisposition to POPF is essentially unmodifiable, and no technical factor in isolation has been shown to overcome unfavourable gland biology. Nevertheless, a more individualised understanding of risk and the optimisation of technique based on that stratification have been a focus of much of the literature over the past decade. Importantly, conflicting results across studies of technical strategies largely reflect heterogeneity in POPF definitions, patient risk profiles, surgical techniques, institutional experience and differences in perioperative care over time. Critically, these factors should be considered when interpreting variable findings rather than assuming true equivalence or superiority of any single intervention.

### 4.1. Fistula Risk Score

Numerous fistula risk models have been developed to estimate the likelihood of postoperative pancreatic fistula, with the most widely adopted being the fistula risk score (FRS), developed in 2013 by Callery et al., with the goal of creating a risk prediction tool for clinically relevant POPF following pancreaticoduodenectomy [19]. It is the most widely used and validated assessment tool, evaluating intraoperative variables such as gland texture, underlying pathology, main duct diameter, and estimated intraoperative blood loss. Despite being the most validated risk-scoring system, subsequent studies have demonstrated variability in performance across institutions and patient populations, with one study suggesting no correlation of fistula with intraoperative bleeding [20], and another study finding that despite suggestions of robust validation, one limitation is that variability in technical strategies used for the high-risk patient by different surgeons in the studies leads to a reduction in generalisability and risk prediction accuracy [21]. This has therefore driven the development of many derivative models, each attempting to refine predictive accuracy and utility in clinical practice.

In 2019, the alternative FRS (a-FRS), proposed by Mungroop et al., replaced blood loss and pathology with body mass index (BMI) and gland texture, aiming to reduce bias in some cohorts [22]. Then, in 2021, the same group updated the a-FRS (ua-FRS) to include male sex, reflecting its independent association with POPF [23]. The preoperative FRS (preFRS) was developed in 2022 by Kolbinger et al. and added preoperative imaging-based information into the risk score, with the goal of facilitating preoperative risk stratification. The authors proposed that including this would enable dynamic surgical planning and encourage more individualised technical approaches, thereby mitigating intraoperative modifiable risk factors for POPF [24]. This study, however, was a retrospective design in a single centre and therefore not validated on a larger scale, nor has it proven to result in a reduced rate of POPF. Table 2 outlines a comparison of the POPF risk prediction models.

Although the risk-scoring models report accuracy in predicting clinically relevant POPF (CR-POPF), their ability to specifically predict grade C fistula is significantly worse, reflecting that severe fistula evolution is not only influenced by patient characteristics and intraoperative events, but rather a function of the whole perioperative clinical picture, including postoperative factors and delayed recognition of complications [25]. There are additional factors to consider in appraising these models’ validity and utility. Firstly, subjective evaluations of gland morphology, such as texture, exhibit significant variability across individual surgeons [26]. Another limiting factor is that no model integrates dynamic postoperative physiological parameters, which often provide the earliest signal of deterioration.

From a practical perspective, risk models cannot have utility in reducing POPF, because if there were any technique or system that reliably reduced complications of pancreaticoduodenectomy to low rates in high-risk cases, it would surely make sense to use that technique or system for all patients, irrespective of risk modelling. Therefore, the “individualisation” concept is lacking in real-world applicability. However, most research in pancreas surgery is observational, and case numbers are often small. Despite their lack of clinical utility, risk models may play a valuable role in analysing outcome data, and so they have an important role in research methodology. Furthermore, it is important to note that whilst high-impact strategies to reduce complications should be applied broadly rather than restricted to high-risk patients only, existing fistula risk prediction models may potentially have pragmatic value in specifically and carefully selected contexts. These tools may support risk-adjusted postoperative surveillance, for example, the intensity level of monitoring, imaging thresholds, or drain management, and therefore may have beneficial impact in resource allocation or benchmarking across centres with variable case mix. Nevertheless, such applications should only complement, rather than replace, the implementation of evidence-based perioperative systems-level strategies that benefit patients across the risk spectrum.

Interestingly, there is emerging evidence to suggest there are ways we can accurately make early POPF predictions, with evolving biomarkers such as drain amylase kinetics demonstrating promising results. AlMasri et al., in 2024, suggested that the risk of CR-POPF after PD can be accurately estimated using drain fluid amylase levels, and in addition, they proposed a kinetics calculator to facilitate postoperative risk stratification [27]. Furthermore, C-reactive protein (CRP) levels have been shown to potentially predict the development of POPF [28], therefore demonstrating that there are areas for future research into factors that will aid in the accurate prediction of POPF.

### 4.2. Anastomoses in Pancreaticoduodenectomy

Pancreaticojejunostomy (PJ) and pancreaticogastrostomy (PG) remain the two principal methods of reconstruction after pancreaticoduodenectomy. Wang et al. performed a network meta-analysis of sixteen randomised controlled trials including more than 2000 patients and found no significant differences in PJ and PG in the prevention of postoperative pancreatic fistula, overall morbidity, or mortality [29]. Similarly, a position statement by the ISGPS indicated that no specific anastomotic technique eliminates the development of a clinically relevant POPF [30]. However, a more recent network meta-analysis which compared five techniques in fifteen randomised controlled trials (RCTs) demonstrated that duct-to-mucosa PG was the best ranked technique overall, as it was associated with the lowest rates of clinically relevant POPF and had the best outcome profile [31]. Despite this, to date, there is no universal consensus on the anastomotic technique in PD, reflecting the complexity of reconstruction choices; therefore, the most appropriate anastomosis is best guided by surgeon comfort and institutional experience.

There are nuances to the specific anastomotic techniques used either in PJ or PG. In particular, duct-to-mucosa and invagination techniques have been compared in multiple RCTs, with neither method demonstrating efficacy in reducing the rates of clinically relevant POPF [32]. Multiple other studies have indicated that there is no single superior technique [4,33]. Therefore, an individualised approach specifically to anastomosis may be more effective; duct-to-mucosa may favour dilated ducts, whereas invagination may provide a broader interface for soft glands; however, outcomes depend on multiple factors, including tissue handling, vascular preservation, and tension-free approximation.

### 4.3. Stenting

Pancreatic duct stenting remains controversial. Internal duct stents are placed in the main pancreatic duct, and the anastomosis is formed over this, whereas external stents are exteriorised through the proximal jejunum via an enterotomy and through the abdominal wall to drain externally [4]. The rationale for stent placement lies in both decompression and a proposed reduction in trypsin flow across the anastomosis, thereby avoiding corrosion [34]. On the other hand, there are risks involved with stenting, including tube-related complications such as migration and obstruction, and loss of digestive fluids, leading to impaired absorption [32]. A meta-analysis from 2013 evaluated four RCTs and reported that external stenting can decrease the POPF rate [35]. This was further supported by a recent meta-analysis evaluating fifty-five studies and 7512 patients; it reported that external drainage was associated with reduced rates of clinically relevant fistula. In contrast, internal stents have not shown a reliable benefit [36]; however, one meta-analysis reported that in direct comparison, there was no significant difference in postoperative complications between internal and external stenting, and in fact, it suggested that internal stenting may be more favourable [37]. Therefore, there is no universally agreed upon technique that definitively reduces the risk of POPF, thus reflecting the need for future RCTs in this area.

## 5. Pharmacological Agents

The use of pharmacological agents as adjuncts to reduce complications after pancreatic resection includes primarily analogues of somatostatin, steroids, and antibiotics. Somatostatin analogues (SAs) have two proposed beneficial mechanisms: firstly, direct inhibition of acinar cells; and secondly, indirect inhibition of gastrin [38]. The effect is hypothesised to result in reduced flow of enzymes through the pancreatic anastomosis after PD [39].

A recent systematic review and meta-analysis (2023) reported a significantly lower incidence of POPF with SA. However, not only was this conclusion based on low certainty due to the poor quality of the evidence, but there was also no significant difference in mortality or other complications reported with or without SA [38]. Consensus recommendations from the international enhanced recovery after a surgery group reported similar conclusions, suggesting only moderate evidence for a reduction in the severity of POPF with prophylactic SA, without a significant improvement in mortality rates [39]. One limitation in understanding the utility of SA use is that a large proportion of the literature does not differentiate clinically relevant from biochemical POPF. This, in conjunction with the lack of evidence for a significant difference in other complications or mortality, underpins why prophylaxis with SA has not been widely adopted in practice, and the routine use of SA as prophylaxis is not recommended, as results of clinical trials have not yet been validated [40].

Almost all data on the prophylactic use of SA relate to Octreotide [41,42]. Pasireotide is an SA with a longer half-life and a broader binding profile, and has been used in a landmark RCT, where it was associated with a decrease in the rate of clinically significant POPF, leak, and abscess [43,44]. A more recent systematic review and meta-analysis using prophylactic Pasireotide, however, found no evidence of reduction in clinically relevant POPF or improvement in other postoperative complications [45].

The use of perioperative corticosteroids has been investigated due to their potential to attenuate the inflammatory response to major abdominal surgery. A recent systematic review and meta-analysis evaluated five studies involving more than 1400 patients, comparing perioperative corticosteroids to no steroids [46]. The authors found that in PD, there was no benefit with the use of perioperative corticosteroids, reporting no reduction in postoperative complications, and therefore indicating little utility in their use perioperatively other than to reduce postoperative nausea and vomiting. Furthermore, despite the limited evidence of any benefit with either SA or corticosteroids in reducing postoperative complications after pancreatic resections, Pasireotide has been compared directly to Hydrocortisone in a recent randomised clinical trial [47]. In this trial, the only significant difference reported was a lower comprehensive complication index in the Pasireotide group for distal pancreatectomy only; however, there was no overall difference between Pasireotide and Hydrocortisone in the evaluation of clinically relevant POPF. Therefore, as with Octreotide, there are no guidelines suggesting the routine use of Pasireotide or Corticosteroids as a prophylactic strategy after PD.

The use of prophylactic antibiotics remains a cornerstone of perioperative care in major abdominal surgery. Recent high-quality evidence has challenged older narrow-spectrum approaches, with a large randomised clinical trial finding a statistically significant difference in the percentage of patients with 30-day postoperative surgical-site infections when comparing Piperacillin–Tazobactam to standard care [48]. A recent systematic review and meta-analysis (2025) analysed twelve studies, including one RCT and more than 12,000 patients, comparing broad-spectrum antibiotic prophylaxis to standard care in PD. The authors found that broad-spectrum Penicillin-based antibiotic prophylaxis, in comparison to Cephalosporins, resulted in fewer CR-POPF, reduced the rate of postoperative infectious complications and unplanned reoperations, and was associated with lower mortality [49]. The authors suggested that such compelling data should lead to the adoption of broad-spectrum antibiotic prophylaxis as the new gold standard for patients undergoing PD. Similarly, another recent meta-analysis involving 2382 patients who underwent PD supported the implementation of Piperacillin–Tazobactam as standard surgical prophylaxis in current practice, with results demonstrating significantly lower risks of postoperative surgical-site infections, major surgical complications, CR-POPF, and mortality [50].

It is important to note, however, that despite the associated reductions in surgical-site infections and intra-abdominal complications after PD when using Piperacillin–Tazobactam as prophylaxis, its use is not without potential concerns. Routine use of broad-spectrum agents may contribute to antimicrobial resistance and promote selection of multidrug-resistant organisms at both institutional and population levels [51]. In addition, Piperacillin–Tazobactam is more costly than traditional prophylactic regimens, which has negative implications for health system budgets, particularly in high-volume centres [52]. These factors highlight the need to balance potential clinical benefit against stewardship principles and economic impact.

The role of routine antifungal prophylaxis after PD remains uncertain and appears closely linked to preoperative biliary drainage. Recent data suggest that fungal biliary contamination is common in those patients who had preoperative biliary drainage, and that targeted antifungal prophylaxis in this subgroup may reduce infectious complications after PD [53,54]. In contrast, there is little evidence to support routine antifungal use in those patients having PD without preoperative biliary drainage [55]. Overall, when evaluating the literature, the use of antifungal prophylaxis in PD favours a risk-stratified approach, tailored to the presence of preoperative biliary drainage and individual patient risk factors, representing an area in need of future research.

Finally, another pharmacological modality that has demonstrated an association with reduction in complications after PD is chemical thromboprophylaxis. Within early recovery after surgery (ERAS) pathways, this is typically delivered as low-molecular-weight heparin (LMWH), commenced perioperatively alongside early mobilisation and mechanical compression, with many centres extending prophylaxis for up to four weeks after discharge in patients undergoing resection for malignancy or those at increased thrombotic risk. Despite limited evidence in this area, observational analyses have shown less postoperative venous thromboembolism after PD when using chemical prophylaxis [56]. Furthermore, ERAS guidelines from 2019 strongly suggest chemical prophylaxis be started two to twelve hours before commencement of surgery and continued throughout the hospital admission [39].

## 6. Perioperative Nutrition in Pancreas Surgery

PD involves visceral resections and reconstruction of the digestive tract, causing a significant impact on metabolism, digestive and absorptive function, and overall nutritional status. These factors are specifically affected by catabolic stress potentiated by the surgery itself, gland loss, and variable reconstructions [57]. Importantly, malignancy and other pancreas-related pathologies affect both the endocrine and exocrine functions of the gland. Pancreatic exocrine insufficiency manifests as weight loss and steatorrhoea, whereas endocrine insufficiency may manifest as diabetes mellitus. Given that a patient’s nutritional status is likely a determinant of outcomes, malnutrition, sarcopenia, and micronutrient deficiency have been a focus of study in pancreatic surgery [58].

Malnutrition and muscle loss are associated with postoperative morbidities such as infection, CR-POPF, delayed gastric emptying (DGE), time to recovery, and failure to rescue [59]. A position paper published by ISGPS suggested routine preoperative measurement of a patient’s nutritional status, as well as documenting weight loss, BMI, and muscle mass, as these are strong predictors of poor short- and long-term outcomes [60]. Therefore, optimising the perioperative nutritional strategy may significantly improve outcomes after PD.

The currently available perioperative nutrition options include immunomodulating nutrition; total or partial parenteral nutrition; tube-based feeding, including nasogastric, nasojejunal, or jejunostomy; and oral intake. Preoperatively, nutritional optimisation aims to improve physiological reserve and immune competence prior to surgery, and there is evidence to suggest that a combination of preoperative enteral nutrition with immune-nutrient supplements is associated with reduced infectious complications and a lower rate of POPF [61]. In addition, a study that reviewed publications over a period of two decades outlined that patients with unintentional weight loss, hypalbuminaemia, or sarcopenia are likely to benefit most from aggressive nutrition supplementation [62]. It is important to note, however, that aggressive preoperative nutritional supplementation may be constrained by multiple factors, including malignancy-related anorexia, obstructive symptoms, systemic therapy side effects, and patient compliance, and therefore the results may be variable. Immunomodulating nutrition supplements aim to use nutrients to modulate the inflammatory response, with the goal of counteracting postoperative immune impairment. However, the evidence regarding their efficacy is limited, and to date, there is no strong recommendation regarding immune nutrition in patients undergoing PD [63].

When comparing enteral (EN) to parenteral nutrition (PN), the proposed benefits of EN include preserved mucosal barrier integrity, improved immune-modulated defence against bacterial translocation, and modulation of inflammatory responses [64]. Therefore, in the postoperative setting, early restoration of EN is preferred over PN [65]. A Cochrane analysis compared total PN with various forms of EN, including nasojejunal (NJ) and jejunostomy feeding, as well as per-oral intake, after PD [65]. Jejunostomy feeding resulted in a reduced hospital stay; however, there was no convincing evidence to suggest a difference in postoperative complications such as POPF, bleeding, or DGE. However, total PN, compared with NJ feeding, was found to potentially have slightly lower rates of POPF, but little-to-no difference in length of hospital stay or other complications. Oral intake, compared to jejunostomy feeding, was reported to potentially have little-to-no difference in length of hospital stay or postoperative complications, with the authors concluding that there was a significant limitation in the number of randomised trials in this area and suggesting a need for further high-quality evidence.

Current international consensus guidelines, including the ISGPS position statement, support early oral intake within enhanced recovery frameworks [60]. This recommendation, however, suggests that in cases of severe postoperative complications or poor oral intake, supplementary nutrition is paramount, and the preferred method, whenever possible, is EN. Universally instituting early resumption of oral intake after pancreas surgery is, however, most difficult in patients who are experiencing complications and, thereby, likely have the greatest need. The position statement, as well as other guidelines, acknowledge this, indicating that special caution should be used when deciding on oral intake in patients with a CR-POPF [60,65]. Therefore, whilst early oral intake remains an important objective after pancreas surgery, rigid enforcement within an ERAS protocol may be counterproductive in some patients, highlighting the need for an individualised and pragmatic approach.

A recent prospective cohort study evaluated the impact of an individualised perioperative nutritional intervention on the outcomes of patients undergoing pancreas surgery within the ERAS framework. The authors concluded that personalised nutritional interventions improved nutritional status and reduced the length of hospital stay [59]. In addition, the concept of whole nutrition management (WNM) has been proposed. WNM “is a systematic, standardised, and individualised approach to nutrition management…involving dynamic adjustment of nutrition intervention according to the patient’s disease status, nutritional status, demand and intake from admission to discharge” [66]. This single-centre randomised controlled trial reported promising results, positing that WNM was associated with fewer postoperative complications and seemingly a favourable strategy for patients having a PD [66].

Pancreatic enzyme replacement therapy (PERT) is a critical component of postoperative care following PD. Pancreatic exocrine insufficiency is not uncommon after PD, and the consequences of malabsorption, weight loss, and micronutrient deficiency can be debilitating for patients and significantly impair effective postoperative recovery. Moreover, many patients develop pancreatogenic diabetes after resection, which compounds nutritional vulnerability and necessitates coordinated metabolic management alongside exocrine replacement. The Australasian Pancreatic Club recommends routine initiation of PERT following PD and continuation for at least six months postoperatively to mitigate the clinical consequences of pancreatic exocrine insufficiency and prevent significant nutritional derangements [67]. Consistent with this, the American Gastroenterological Association recommends administration of PERT during meals to optimise mixing with ingested nutrients, with this strategy demonstrating reduction in steatorrhoea and overall improvements in nutritional status [68]. Randomised evidence further supports the clinical value of PERT, demonstrating significant improvements in body weight and nutritional parameters following PD when therapy is appropriately prescribed and adhered to [69]. Collectively, these data underscore that optimal postoperative nutritional care after PD requires an integrated approach that combines proactive management of pancreatogenic diabetes with routine, meal-based PERT delivered within a multidisciplinary pathway, aiming to preserve lean body mass, support glycaemic stability, and enhance functional recovery.

Overall, perioperative nutrition in pancreatic surgery represents a significant challenge. Patients at greatest risk of complications preoperatively and those who actually experience complications postoperatively face the greatest barriers to optimal nutritional strategies. There is a strong biological and physiological rationale for the importance of perioperative nutritional optimisation in pancreas surgery; however, limited evidence guides practice due to heterogeneity in study designs, limited generalisability, and small sample sizes, reflecting the imperative for future research in this area.

## 7. Systems-Based Approaches—Centralisation, Failure to Rescue, Algorithm-Based Management and Care Protocols

Given the complex nature of PD and the high risk for potentially disastrous complications, systems-based strategies are essential drivers of improved outcomes. Such strategies include centralising pancreas surgery, implementing algorithm-based perioperative management, and using institutional protocols with tools to aid compliance.

There is clearly an association between centre volume and outcomes such as mortality and cost in pancreas surgery [70,71,72,73,74,75]. This has led to an impetus toward mandated centralisation. Whilst these data are interesting, two important anomalies remain unresolved. First, centralisation policies are often proposed using minimum-volume standards with a lower bound for acceptable volume (as low as 5–10 cases per year), so they have little biological or technical credibility [76,77]. Second, the marginal benefits of additional volume as centres grow from medium-to-low to high volume are hard to demonstrate, suggesting a very narrow range of benefits associated with centre volume [78,79,80]. Centralisation is built on the notion that the provision of complex procedures such as PD is better organised in such a way that fewer specialist units serve a larger number of patients [76]. Policy intervention to divert pancreatic surgical procedures to fewer specialised centres relies on the implicit hypothesis that greater case volume leads to superior outcomes [81,82,83]. However, all these data are observational, and so causation is unclear. An equally plausible explanation for this observation is the outcome-to-volume hypothesis, which proposes that centres with superior service provision, resources, and outcomes attract a larger number of patients, thereby increasing volume [82,84]. Whilst this may seem like a distinction without a difference, the implication of the arrow of causation may be significant if policy intervention is based on the assumption of the causal relationship of volume on outcome. Policy interventions to increase centre volume may not produce the anticipated benefits if the policy intervention is not accompanied by the resources (mostly institutional infrastructure and experienced personnel, which allow for successful rescue). Even if undertaken purely for health economic imperatives, centralisation could have negative impacts on the population, particularly in large or sparsely populated service areas. Service centralisation may lead to increased costs and inconvenience for patients and their families. If patients do not access surgical care, this effect cannot be discerned from analysis of surgical outcomes, but only through population disease-related metrics. Indeed, there is a strong association between rurality and inferior pancreas surgery rates and cancer-specific survival [85,86,87]. When rural patients access care, they seem to have similar outcomes to urban patients, suggesting that it is the access to care rather than some other factors which drives this disparity [88]. This means that improvement in surgical outcomes through centralisation may represent a trade-off with a detriment in cancer outcomes through inferior access to surgical care for some patients, particularly those from rural areas in large and sparsely populated jurisdictions.

Despite the clear association between fewer complications and higher-volume centres, it is important to highlight that the superior outcomes in larger centres may not be specifically related to lower complication rates, but rather a lower rate of death in patients who experience a complication [77,88,89]. Originally described by Silber in the 1990s [90], failure to rescue is defined as death following a major postoperative complication. It encapsulates the contributions of systems and the entire range of medical and allied health staff in managing surgical complications before they become irreversible and ultimately fatal [91]. Failure to rescue has increasingly become a metric of quality, perhaps more important than mortality. One reason for this is that surgical complications are strongly associated with patient factors and therefore not preventable other than by more stringent patient selection [92]. Therefore, whilst volume thresholds may be used to define eligibility to be a pancreas surgery centre, volume alone may not suffice as a surrogate for institutional capability. Using failure-to-rescue metrics likely provides a more meaningful measure of system performance, particularly when evaluating the impact of centralisation.

The rate of failure to rescue varies significantly between institutions, as well as between geographical regions, and is itself influenced by multiple factors, including patient factors such as the American Society of Anaesthesiologists (ASA) class, patient age, the presence of ascites and/or varices, and disseminated malignancy [93,94]. In addition, institutional factors, such as the presence of surgical trainees and medical students, have been associated with reduced odds of failure to rescue [94,95]. Although the benefits of less experienced practitioners seem paradoxical, trainees and students are hypothesised to see patients more frequently, have fewer competing demands, and thus recognise subtle physiological deviations more readily. In addition, trainees’ situational awareness, comfort in escalating concerns, and lack of ingrained cognitive biases during post-operative assessments may be responsible for the association between trainees and teaching environments and a reduced rate of failure to rescue.

**Figure 2:**
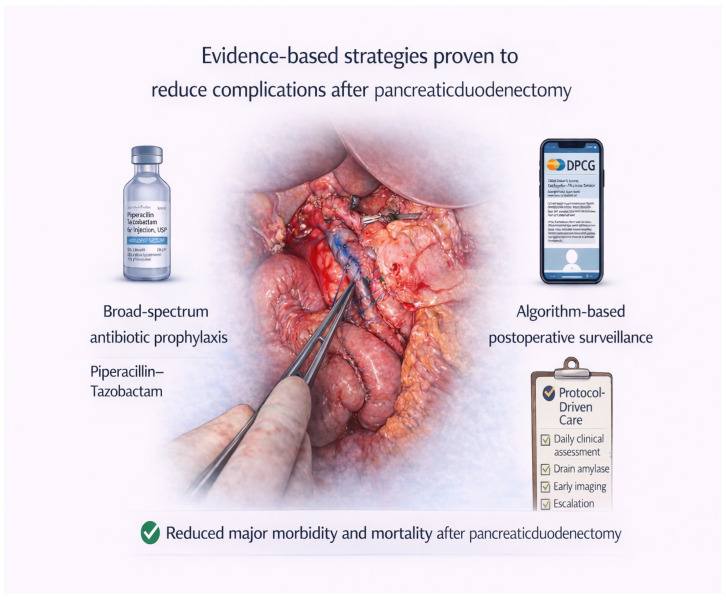
Evidence-based strategies to reduce complications after PD. Visual summary highlighting two interventions supported by high-level evidence: broad-spectrum antibiotic prophylaxis with Piperacillin–Tazobactam and algorithm-based postoperative surveillance (PORSCH), both associated with reduced major morbidity and mortality following PD.

The importance of early recognition of objective deterioration, avoidance of cognitive bias, and the rapid institution of treatment has been further highlighted by the concept of algorithm-based care. The PORSCH trial is the most compelling evidence in this domain [96]. This nationwide, stepped-wedge cluster RCT included all seventeen centres that perform pancreatic surgery in the Netherlands over a 22-month period. During the study, each centre crossed over from “usual care” to “algorithm-based care” at a randomised timepoint. The intervention comprised a smartphone application that would guide clinicians in the daily evaluation of clinical, biochemical, and radiological criteria from postoperative day three onward. The primary composite endpoint was bleeding requiring invasive intervention, new-onset organ failure, or 90-day mortality. The application used an algorithm that specified thresholds for interventions, including imaging of patients, commencing antibiotic therapy, or intervention with percutaneous drainage. The results of the PORSCH trial were striking, with the authors concluding that early recognition and minimally invasive management through the use of a novel protocolled algorithm markedly improved outcomes in patients who had pancreas resections compared to usual care, with an overall reduction in mortality of up to fifty percent [96]. The reported outcomes in this trial support the use of algorithm-based care to identify and treat complications before they negatively impact a patient’s clinical outcome. Interestingly, another important mechanistic feature highlighted in this trial was that minimally invasive intervention reduced the need for salvage relaparotomy. This overall reduction in the failure-to-rescue rate, without reducing the overall incidence of complications after pancreas resections, thereby emphasises the importance of focusing on the rescue element over prevention of complications altogether. Figure 2 depicts evidenced-based strategies to reduce complications after PD. There are, however, some important factors to consider when evaluating the PORSCH trial and its real-world application. First, this study was conducted in the Netherlands, which has a centralised system for pancreas resections with a uniform audit infrastructure, which may affect generalisability outside that country. Second, compliance with the smartphone application varied, and therefore, real-world implementation of algorithm-based care may pose challenges outside of a clinical trial. Finally, protocol-based rigidity may reduce clinicians’ flexibility in assessing and acting expeditiously in the face of atypical patient trajectories and presentations.

In addition to the PORSCH trial, other studies have demonstrated the positive impact of perioperative algorithm-based care and clinical pathways on the management of patients undergoing complex pancreas surgery. Van Der Kolk et al. (2017) concluded that the use of a clinical pathway in pancreaticoduodenectomy patients was associated with improved outcomes [97], suggesting that a standardised care plan resulted in fewer complications, shorter length of hospital admission, fewer readmissions, a reduced rate of gastroparesis, and shorter time to enteral feeding and ambulation [98,99,100]. Furthermore, ERAS programmes specific to PD have also demonstrated promising results in reducing the rate of complications and the length of hospital admission, while maintaining patient safety through early mobilisation, early oral feeding, and optimised pain control [101].

Implementation of algorithm-based care, however, is likely to be more challenging in low-income populations, where access to high-volume centres, specialised multidisciplinary teams, advanced imaging modalities, and timely interventional radiology may be limited. Workforce constraints, fragmented referral pathways, and variability in critical care resources can further impede consistent protocol adherence and expeditious escalation, potentially blunting the benefits of structured pathways. Nonetheless, emerging digital health and telemonitoring approaches offer potential to partially mitigate these barriers. Remote physiological monitoring, mobile-based early warning tools, and virtual specialist review may facilitate earlier recognition of deterioration and support decision making in resource-constrained settings [102]. Whilst such technologies still require rigorous evaluation, they represent a promising adjunct to algorithm-based care models in the future.

Moreover, when evaluating studies reporting outcomes of algorithm-based care, it is important to be cognisant of other certain limitations. Except for the PORSCH trial, most algorithm-based care studies are small, inadequately powered, retrospective, and observational, with a high risk of bias, confounding, and type 2 error. Many of these studies were performed in high-volume, well-resourced institutions, which foster a culture of quality improvement, and therefore the results may not be generalisable outside of this context. Despite these factors, there is enough evidence to suggest that algorithm-based care in complex pancreas surgery can reduce mortality, and a future focus on randomised studies in this domain would be beneficial.

## 8. Future Directions

In recent years, pancreatic surgery and perioperative care have evolved in the context of technical advancement, multidisciplinary models of care, and an improved understanding of the pathophysiological factors driving complications. Near-term anticipated developments include ERAS protocols incorporating immunomodulated nutrition, prehabilitation, radiomics in the diagnosis of pancreatic lesions, intraoperative imaging and Indocyanine Green (ICG), and augmented reality with surgical robotics. The adoption of ERAS protocols, including optimised perioperative nutrition and pharmacological management, has shown promise in improving recovery times and reducing complications [103]. These protocols emphasise the early resumption of oral intake, the use of immunomodulated nutrition, and proactive management of pancreatic exocrine insufficiency [40]. Radiomics involves extracting quantitative, reproducible data from radiological images [104]. It capitalises on advanced image analysis to characterise tumour phenotypes, define pancreatic pathology more reliably, and discriminate between benign and malignant tumours [105,106]. Intraoperative near-infrared imaging using ICG has been shown to allow both real-time analysis of tissue perfusion and margin assessment [107].

There has been an increasing use of minimally invasive and robotic surgical techniques in pancreatic resection over the past few decades. Although laparoscopic pancreaticoduodenectomy was first performed in the 1990s, its adoption has been slower than that of other intra-abdominal laparoscopic procedures, owing to its technical complexity, steep learning curve, and prolonged procedural times [108,109,110]. Robotic surgery, on the other hand, offers advantages over laparoscopic minimally invasive techniques due to the enhanced dexterity, high-definition three-dimensional vision, and tremor filtration offered by the robotic platforms [111]. However, outside of short-term recovery, there is limited evidence that laparoscopic or robotic techniques offer any advantage in the avoidance of the major complications in pancreas surgery caused by POPF, and they are consequently far from replacing open surgery, particularly in PD [89]. Notably though, robotics is a fertile area for research, particularly in combination with other emerging technologies, such as augmented reality and 3D printing [112].

Looking ahead, several areas warrant focused investigation to further reduce complications after PD. First, there is a clear need for well-designed, validated multicentre randomised trials of systems-based interventions, including algorithm-driven pathways, escalation protocols, and failure-to-rescue strategies, in order to determine which organisational model most effectively improves outcomes across diverse healthcare settings. Secondly, the integration of artificial intelligence and radiomics-based preoperative risk stratification offers a promising avenue to refine patient selection, anticipate complications, and individualise perioperative strategies. This, however, requires prospective validation and standardisation before routine clinical adoption. Finally, the development of global benchmarking frameworks for failure to rescue, that is, the incorporation of risk-adjusted metrics, transparency in reporting, and cross-institutional learning, may facilitate continuous quality improvement and more equitable outcomes in pancreatic surgery worldwide.

Future work should prioritise approaches that move beyond risk prediction to actionable changes in care, such as embedding imaging-based risk stratification into operative planning and postoperative surveillance pathways. Equally, strengthening failure to rescue will depend on implementing standardised monitoring, trigger-based escalation models, and digital early-warning systems within high-volume networks to ensure timely and consistent responses to deterioration

## 9. Conclusions

Complications following pancreaticoduodenectomy remain a major barrier to improving outcomes. Postoperative pancreatic fistula continues to drive much of the morbidity and mortality associated with the surgery. Despite sustained technical advances and refinements in perioperative care, no single operative strategy has reliably eliminated the risk of major complications. Increasingly, evidence suggests that meaningful improvements in outcomes are more likely to be achieved through coordinated perioperative frameworks rather than further isolated technical modifications. Among the wide range of interventions evaluated, two strategies seem to be supported by high-level evidence. Broad-spectrum antibiotic prophylaxis with Piperacillin–Tazobactam has been shown to reduce infectious complications and clinically relevant postoperative pancreatic fistula, with emerging evidence of reduced mortality. In parallel, algorithm-based postoperative surveillance and escalation pathways, as demonstrated in the PORSCH trial, provide strong evidence that protocol-driven care embedded within enhanced recovery frameworks can significantly reduce major complications and death. Together, these findings emphasise that optimising postoperative systems of care and early rescue capability may exert a greater influence on survival than further modification of anastomotic techniques. By contrast, most other technical, pharmacological, and nutritional strategies remain supported by heterogeneous and often conflicting evidence. This uncertainty highlights a critical need for rigorously designed and adequately powered multicentre trials.

## Figures and Tables

**Table 1:** Non-modifiable and modifiable factors associated with prediction of postoperative pancreatic fistula.

Patient		Pancreatic		Procedure		Pathology		Systems	
NM	M	NM	M	NM	M	NM	M	NM	M
Male sex	Obesity	Small main duct	Soft gland texture	Type of procedure (PD)	Centre volume	Benign disease			Drain placement and management
	Malnutrition/sarcopenia		Poor parenchymal vascularity on CT		Surgeon experience	Cystic lesions			Structured leak pathways
	Steroid exposure				Intraoperative blood loss	Neuroendocrine tumours			Somatostatin analogues
	Anticoagulation				Technical approach and reconstruction				Steroid use
									Centralisation of care
									Failure to rescue and protocol-driven care

NM = non-modifiable; M = Modifiable.

**Table 2:** Comparative postoperative pancreatic fistula (POPF) risk prediction models used in pancreatic surgery.

Model	Target Procedure	Key Variables Included	How Risk Is Expressed	Main Strengths	Key Limitations
Original FRS (Callery)	PD	Gland texture, duct size, pathology, blood loss	4-tier categorical score (negligible → high risk)	Simple, widely validated; intuitive for surgeons	Intraoperative; limited guidance on how to modify risk
a-FRS/ua-FRS (Mungroop)	PD (open and minimally invasive)	BMI, gland texture, duct size, pathology	Continuous probability estimate	Better discrimination than original FRS in some cohorts	Still largely descriptive rather than prescriptive
preFRS (Kolbinger)	Pancreatic head resection	CT-based radiomic features	Preoperative probability score	Allows preoperative risk stratification	Requires specialised imaging analysis

## Data Availability

No new data were created or analysed in this study. Data sharing is not applicable to this article.

## Abbreviations

The following abbreviations are used in this manuscript:

PD	Pancreaticoduodenectomy
POPF	Postoperative pancreatic fistula
ISGPS	International study group on pancreatic surgery
FRS	Fistula risk score
A-FRS	Alternative fistula risk score
BMI	Body mass index
Ua-FRS	Updated alternative fistula risk score
CR-POPF	Clinically relevant postoperative pancreatic fistula
CRP	C-reactive protein
PJ	Pancreaticojejunostomy
PG	Pancreaticogastrostomy
RCT	Randomised controlled trial
SA	Somatostatin analogue
ERAS	Early recovery after surgery
LMWH	Low-molecular-weight heparin
DGE	Delayed gastric emptying
EN	Enteral nutrition
PN	Parenteral nutrition
NJ	Nasojejunal
WNM	Whole nutrition management
PERT	Pancreatic enzyme replacement therapy
ASA	American Society of Anaesthesiologists
ICG	Indocyanine Green
